# A 2.5D Map-Based Mobile Robot Localization via Cooperation of Aerial and Ground Robots

**DOI:** 10.3390/s17122730

**Published:** 2017-11-25

**Authors:** Tae Hyeon Nam, Jae Hong Shim, Young Im Cho

**Affiliations:** 1 Department of Mechatronics Engineering, Graduate School, Korea Polytechnic University, Si-Heung City 15073, Korea; namtae91@gmail.com; 2 Department of Mechatronics Engineering, Korea Polytechnic University, Si-Heung City 15073, Korea; 3 Department of Computer Engineering, Gachon University, Sung-Nam 13120, Korea

**Keywords:** SLAM, localization, ground robot, aerial robot, cooperation, low cost sensor, indoor

## Abstract

Recently, there has been increasing interest in studying the task coordination of aerial and ground robots. When a robot begins navigation in an unknown area, it has no information about the surrounding environment. Accordingly, for robots to perform tasks based on location information, they need a simultaneous localization and mapping (SLAM) process that uses sensor information to draw a map of the environment, while simultaneously estimating the current location of the robot on the map. This paper aims to present a localization method based in cooperation between aerial and ground robots in an indoor environment. The proposed method allows a ground robot to reach accurate destination by using a 2.5D elevation map built by a low-cost RGB-D (Red Green and Blue-Depth) sensor and 2D Laser sensor attached onto an aerial robot. A 2.5D elevation map is formed by projecting height information of an obstacle using depth information obtained by the RGB-D sensor onto a grid map, which is generated by using the 2D Laser sensor and scan matching. Experimental results demonstrate the effectiveness of the proposed method for its accuracy in location recognition and computing speed.

## 1. Introduction

When a robot begins navigation in an unknown area, it has no information about the surrounding environment. Accordingly, for robots to perform tasks based on location information, they need a Simultaneous Localization and Mapping (SLAM) process that uses sensor information to draw a map of the environment, while simultaneously estimating the robot’s current location on the map. For example, recently commercialized cleaning robots featuring on-board location recognition functions establish the location of the device as a reference point when it is turned on. The robot then draws a map with the information collected from built-in sensors (e.g., vision sensor, distance sensor), while simultaneously estimating its own location in real-time to distinguish between areas that have been cleaned and areas that need to be cleaned.

A variety of sensors, such as distance and vision sensors, are used in SLAM. Distance sensors typically include laser scanners, infrared scanners, ultrasonic sensors, LIDAR, and RADAR, whereas vision sensors include stereo cameras, mono cameras, omnidirectional cameras, and Kinect [1,2,3,4]. Distance sensors can easily obtain distance information about objects, but the type of obtainable data is limited. In contrast, vision sensors, which have recently been in the spotlight, can acquire a variety of information, such as distance and object information, through processing imagery information. However, the majority of the information requires significant computation power. Generally, in the field of mobile robotics, the information obtained by the sensors is integrated with odometry (e.g., encoders, inertia sensors) to be used, but the research on performing SLAM only with only vision sensors has been actively carried out [5,6,7,8,9]. 

Various methodologies have been studied to resolve a variety of issues including the SLAM process under wide environments, uncertainty of sensor observational data, and real time execution. Representative methods are those based on the Kalman filter (KF) [10,11,12], Particle filter (PF) [13,14], graph [15,16], and bundle adjustment (BA) [17]. The KF is difficult to be applied to a nonlinear system as it basically assumes system linearity. Accordingly, the extended Kalman filter (EKF) and unscented Kalman filter (UKF) are primarily used for typical nonlinear systems. The EKF is disadvantageous in its likeliness to be easily diverged under incorrectly designed system, but it demonstrates favorable performance in the fields of current navigation and nonlinear state estimation, such as in GPS. The PF expresses location uncertainties as particle groups, which are referred to as samples. If the number of samples is sufficient, it is more accurate than the EKF or UKF, but issues can arise when the number is insufficient. There are also methods that simultaneously use the PF and KF, with the Rao-Blackwellized particle filter-based SLAM being representative of them.

Recently, SLAM studies are being carried out through cooperation of a UGV (unmanned ground vehicles) and UAV (unmanned aerial vehicles) to actualize more accurate mapping by improving capability in situational awareness and environment interaction [18,19,20].

A UGV may carry substantial payloads and can actively interact with environments. In a search and rescue mission, a UGV can be sent to the location where the settings are too dangerous for humans. It may reduce the time required to reach victims by removing the need to secure the area in the first place. However, the operator only receives limited information about the UGV’s surroundings owing to its low viewpoint. Other objects at the height of the robot may block the operator’s view, increasing the difficulty of navigating within hazardous environments.

UAV, on the other hand, can provide a situational assessment of the environment through its ability to cover large areas quickly with its bird’s-eye view. This data enables global navigation of the UGV in a potentially unknown and challenging terrain. Therefore, working in a heterogeneous team of UAV and UGV enhances the capabilities of the robots to support human rescue teams.

If the map generation and location recognition are performed using the UAV, the entire map can be generated without being significantly affected by the features of land. However, UAV is not suitable to carry out tasks such as lifesaving and obstacle removal, while UGV is. In case of map generation and location recognition, the UGV is considerably affected by the land features and, especially in case of a disaster, it is very difficult to build a useful map owing to the existence of non-driving areas. Therefore, in recent years, researches on the methods of sharing location and map information using features of the two robots have been conducted [21,22,23]. 

Previous work on the collaborative tasks between aerial and ground robots often involved performing the entire perception process on one robot and guiding the other through the mission. For example, a flying robot can track a ground robot using a QR-code [24], visual markers [25,26], or edge detection [27]. Other works used a camera on a ground robot to track the LEDs on a flying robot [28,29]. 

Additionally, many approaches are based on collaborative navigation in large outdoor environments with at least partial availability of GPS [30,31]. Therefore, these approaches are not applicable under indoor scenarios and locations without GPS available, such as forests or urban street canyons. In addition, even if a GPS signal is available, the position is often inaccurate and provides no heading direction. 

On the other hand, methods to globally localize planetary rovers in the absence of GPS exist for space exploration. For this purpose, visually detectable landmarks [32] or generated surface elevation maps of the rover surroundings of the rover are matched to a global map. The elevation maps are therefore searched for topographic peaks [33] or compared directly using a zero-mean normalized cross-correlation [34]. 

In the context of UGV-UAV collaboration, Rudol presents a system paper on a UGV guiding a UAV to navigate in an indoor environment by using a LED cube structure pattern attached to the UAV [35]. In [36], the authors propose a cooperative control framework for real time task generation and allocation for a hierarchical UAV/UGV platform. 

Michael, N. [37] introduced the collaborative mapping method using a 3D laser scanner for investigating the damage caused by an earthquake. However, as it used the point cloud of a 3D laser scanner, vast amount of data led to lengthy map generation. Thus, when it is mounted on a UAV for traveling, it has very high power consumption. Oleynikova, H. [38] proposed real-time localization of a UAV with a UGV using inertial and vision sensors. To synchronize the initial position of the two robots, the UAV performs takeoff and landing at the UGV’s top. Early work on cooperative localization and mapping extended the well-studied single robot SLAM problem to multiple robots. 

Vidal-Calleja, T.A. [39] proposed multiple robot visual mapping with heterogeneous landmarks in GPS-denied and unexplored environments. Images captured from an UGV were matched to those from a satellite or high-flying vehicle. The warped ground images are then matched to the satellite map using descriptor-based feature matching techniques. For feature matching, they used a PF. However, if the data is limited, the result has the drawback of being inaccurate.

Data format changes depending on the type of sensor used to generate a map for cooperation of the two robots. Usually, the majority of 3D sensors, such as the 3D LRF, are very costly. When raw data of 3D point cloud data is used, real-time location recognition and navigation become difficult as volume of the data increases and more computation is needed. On the other hand, when using 2D laser scanner sensors, it takes less time to create a map, but there are instances in which the information needed for the mobile robot’s autonomous navigation is not fully incorporated because the scanner can only recognize obstacles of a certain height. Also, there can be instances in which the scanner recognizes navigable slopes as obstacles as it cannot adequately incorporate height information needed.

In this paper, we proposed a method for accurate map generation and real-time location recognition of a mobile robot in indoor environments by combining the advantages of 3D map and 2D map through cooperation between UAV and UGV. We used an RGB-D (Red Green Blue-Depth) sensor, which is a low-cost 3D sensor, and installed it onto a UAV to generate a 3D map. To reduce computation burden for real-time localization, the 3D map was transformed into a 2D map, and the depth value included in the 3D map was used to estimate heights of obstacles. By including the height information into the 2D map, a 2.5D elevation map was generated. Moreover, to improve the accuracy of the generated 2.5D map, a low-cost 2D LRF sensor was used to propose a method that connects the position value of the 2D LRF with location information obtained by the RGB-D sensor. Through a series of experiments, it was demonstrated that the method using a 2.5D map built by a 2D LRF sensor and a RGB-D sensor installed in an UAV effectively performs tasks of mobile robot’s localization and navigation.

## 2. Comparison of RGB-D and LRF SLAM on UGV

**Figure 1:**
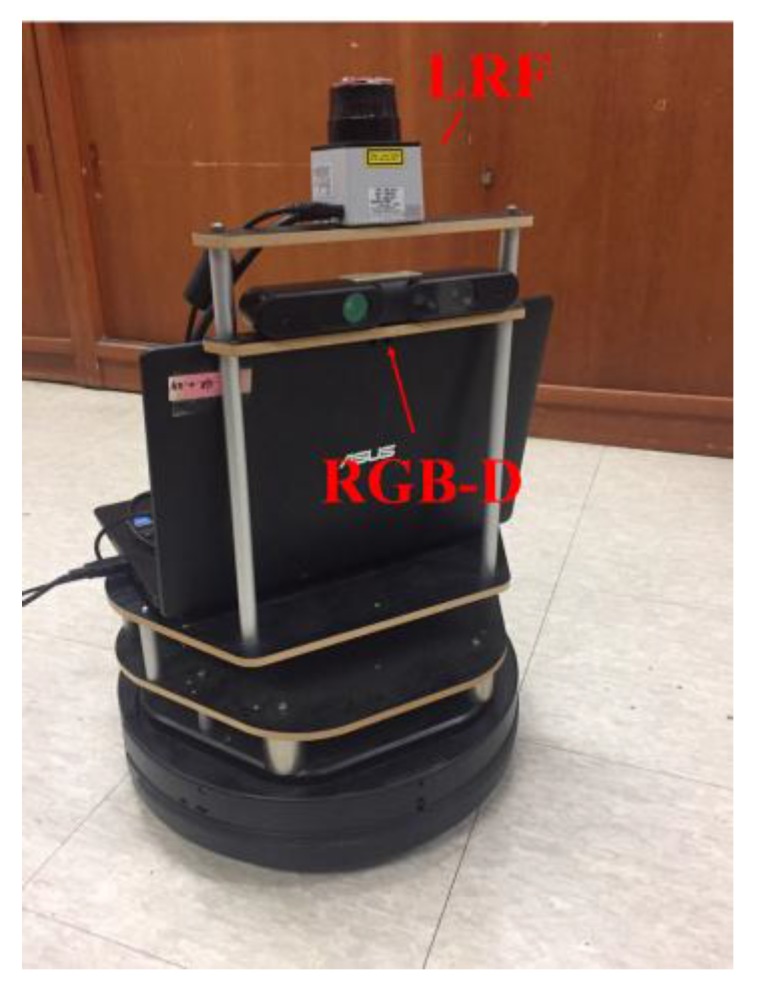
Ground robot used in experiment.

This section describes the implementation of the RGB-D SLAM and LRF SLAM that are usually used for mobile robots in indoor environments, and compares the results of each SLAM method. Figure 1 presents turtlebot.2 from the Yujin Robot, which was used as the mobile robot in the experiment. The height of the robot is 50 cm. A 2D LRF and a RGB-D sensor were installed on the robot. The LRF and the RGB-D sensor were installed at a height of 57 cm and 45 cm respectively. The LRF sensor (URG-04LX from HOKUYO) has the measurable angle of 240 degrees, the distance resolution of 1 mm, and the angular resolution of 0.3515 degrees. The RGB-D sensor has the measurable angle of 57 degrees, the distance resolution of 2.5 to 4.8 mm, and the angular resolution of 0.097 degrees.

**Figure 2:**
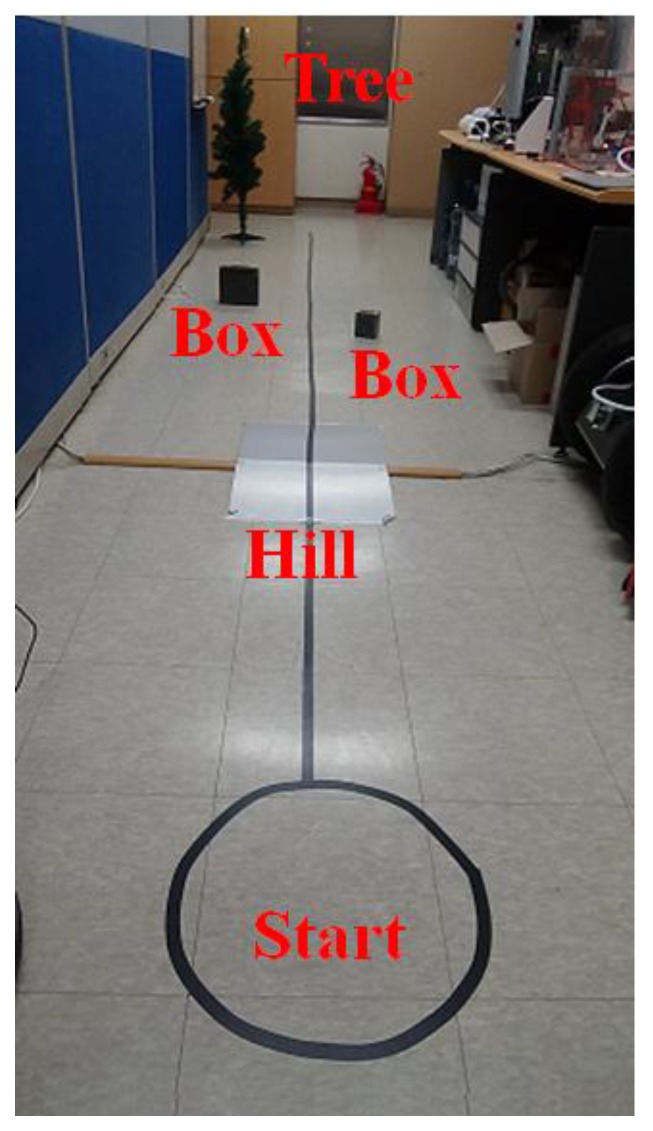
Experimental environment.

Figure 2 shows the designed experimental environment. The robot navigated back and forth on a 10-m section of the straight black line. The location errors were calculated from the difference between its actual location and the location estimated by SLAM after navigation. As shown in the figure, obstacles such as a sloping hill, a tree, and two boxes were installed in the experimental environment. Experiment shown in Figure 2 was carried out in the indoor environment, of which dimensions was: 15 m (length) × 1.5 m (width) × 2.5 m (height). 

This study used the ICP (Iterative Closest Point) algorithm to generate the location recognition and mapping of the robot using sensors. The ICP algorithm generates a map via point-to-point matching. The Euclidean distance in the data measured in real time through sensors and previous data calculates the closest matched pair. The scan matching program using the ICP algorithm must make a one-to-one comparison of N standard data and N new data. During matching process, scan matching is performed if errors of location and rotation are larger than the threshold value. If the errors are smaller than the threshold value and determined to be identical, the scan matching concludes. For scan matching, the rotation matrix (*R*) and translation matrix (*t*) should be found and the error function *E* is calculates as Equation (3) [40,41].

The following are two data sets for scan matching and an error function *E*.

(1) $$X=\{x_{1},x_{2},\ldots ,x_{n}\}\;$$

(2) $$P=\left\{p_{1},p_{2},\ldots ,p_{\;n}\right\}$$

(3) $$E\left\{R,t\right\}=\frac{1}{N_{p}}\overset{N_{p}}{\underset{i=1}{\sum }}{\left\|x_{i}-R\;p_{i}-t\right\|}^{2}$$

Here, $N_{p}$ is the number of data points, and $x_{i},\;p_{i}$ are the matching points. The center of the data set must be found to calculate errors in the data values.

(4) $$\mu_{x}=\frac{1}{N_{x}}\overset{N_{x}}{\underset{i=1}{\sum }}x_{i}$$

(5) $$\mu_{p}=\frac{1}{N_{p}}\overset{N_{p}}{\underset{i=1}{\sum }}p_{i}$$

The data value deviation is calculated from the differences of the center of the data set and differences of data as shown in Equations (6) and (7).

(6) $$X^{\prime }=\left\{x_{i}-\mu_{x}\right\}=\left\{x_{i}^{\prime }\right\}$$

(7) $$P^{\prime }=\left\{p_{i}-\mu_{p}\right\}=\left\{p_{i}^{\prime }\right\}$$

Next, the SVD (Singular Value Decomposition) is used to find optimal solutions for the rotation matrix and translation matrix. Below is the set W for the matching pairs.

(8) $$W=\overset{N_{p}}{\underset{i=1}{\sum }}x_{i}^{\prime }p_{i}^{\prime }{}^{T}$$

(9) $$W=U\left[\begin{array}{ccc} \sigma_{1} & 0 & 0\\ 0 & \sigma_{2} & 0\\ 0 & 0 & \sigma_{3} \end{array}\right]V^{T}$$

Here, U and V are 3 × 3 single matrices, respectively, and $\sigma_{i}$ is the singular value of matrix W that satisfies following conditions. If rank (W) = 3, the optimal solution for E{R,t} is calculated using the formula below.

(10) $$R=UV^{T}$$

(11) $$t=\mu_{x}-R\;\mu_{p}$$

(12) $$E\left\{R,t\right\}=\overset{N_{p}}{\underset{i=1}{\sum }}\left({\left\|x_{i}^{\prime }\|{}^{2}+\|y_{i}^{\prime }\right\|}^{2}\right)-2\left(\sigma_{1}+\sigma_{2}+\sigma_{3}\right)$$

**Figure 3:**
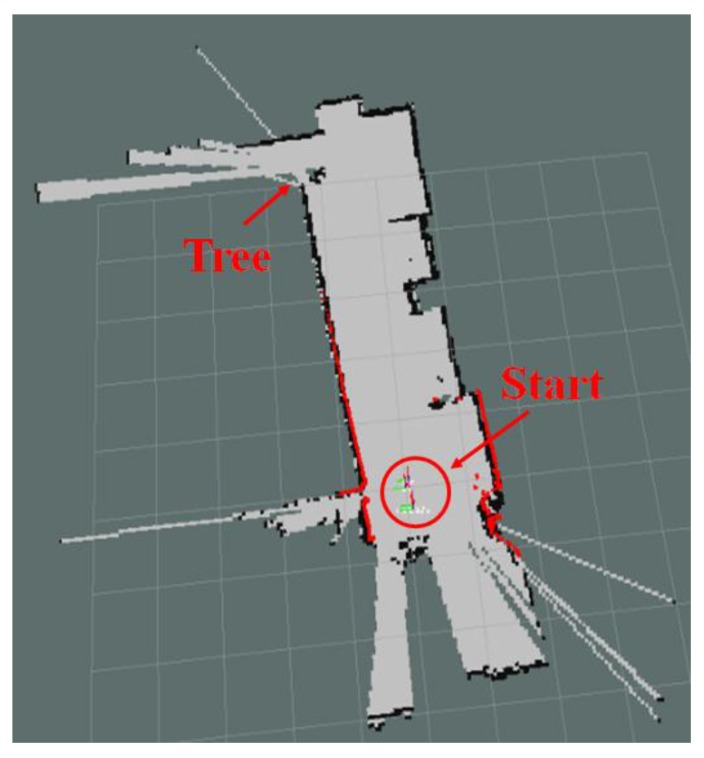
2D Map information generated by using the 2D LRF sensor.

Figure 3 shows the 2D map generated using the ICP algorithm and 2D LRF sensor. Objects over a certain height were recognized as obstacles, but objects of relatively lower height, such as the box, were not recognized as obstacles. As the 2D LRF SLAM uses the mobile robot’s encoder values as odometry and a slope was located on the path during navigation of the 2D LRF, location error of approximately 30 cm on average was observed. Table 1 shows the location recognition errors according to the number of the mobile robot’s round trip along the path of Figure 2.

**Figure 4:**
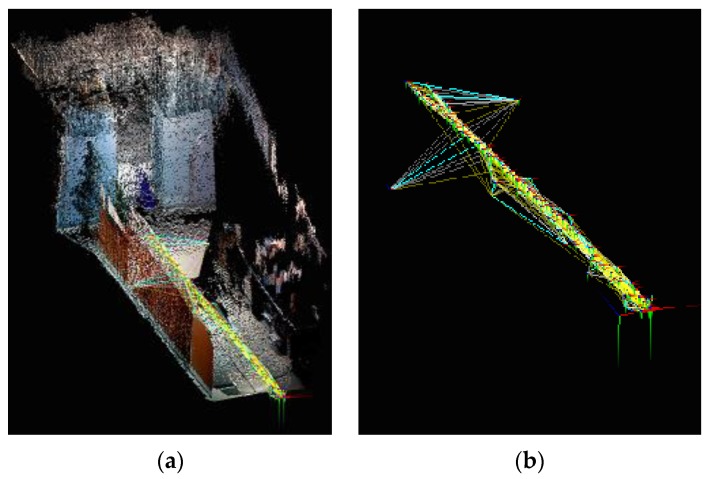
(**a**) 3D Map made by a RGB-D camera of the mobile robot; (**b**) navigation trajectory of the UGV.

For the SLAM using 2D LRF, instances occurred in which the robot was unable to recognize obstacles of a lower height, and the SLAM was performed using an RGB-D camera to overcome this limitation. Figure 4a shows a 3D map generated by using an RGB-D camera installed in the mobile robot under an identical experiment environment to that of Figure 2. Figure 4b shows the results of the navigation trajectory of the mobile robot, which was conducted 10 times under the same experimental environment. Table 2 lists the location recognition errors based on the robot’s navigation trials applying the RGB-D SLAM. The location recognition errors were measured as the differences between the actual location and the estimated location of the robot after SLAM-based navigation.

In case of RGB-D SLAM, the robot was able to recognize obstacles of lower height. Further, as the RGB-D SLAM does not rely solely on the wheel’s encoder information, it was able to reduce errors during the slope navigation by using visual odometry information to correct error. Despite the corrections, however, an error of approximately 5–10 cm occurred due to vibrations of the robot and camera movement during the slope navigation. Moreover, due to the 3D mapping, there was significant data computation increase, and the processing speed was about 3–4 fps (frames per second).

**Figure 5:**
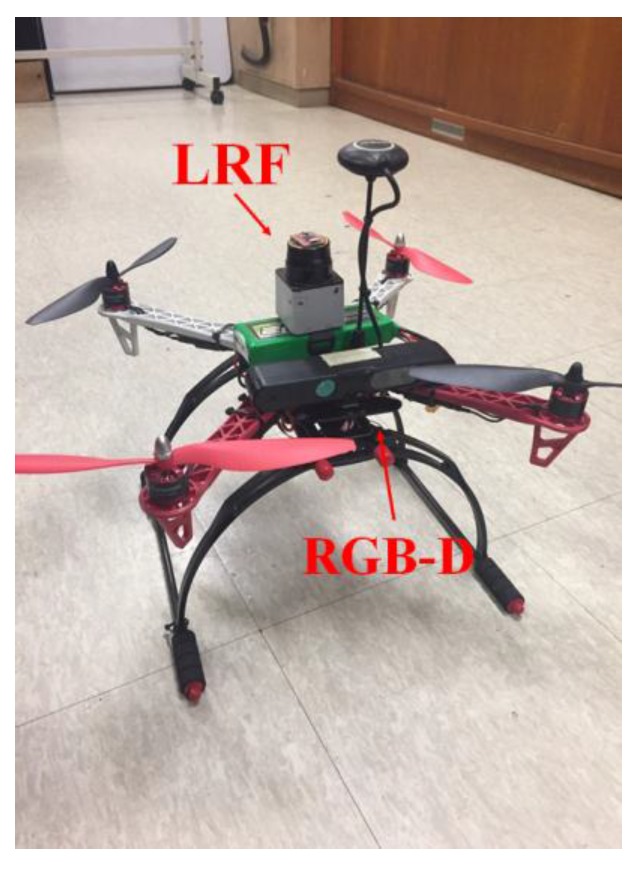
Aerial robot used in this study.

Through the two experiments, this study verified that topography and presence of obstacles cause location recognition errors in the LRF and RGB-D SLAM using a mobile robot. Thus, when using a mobile robot for mapping, a navigation algorithm that allows avoidance of obstacles is absolutely needed. To address these issues, this study performed SLAM using an aerial robot that is virtually unaffected by geographic features, and an RGB-D camera that includes information on various obstacles. Figure 5 shows the DJI F450 series aerial robot used in this study.

**Figure 6:**
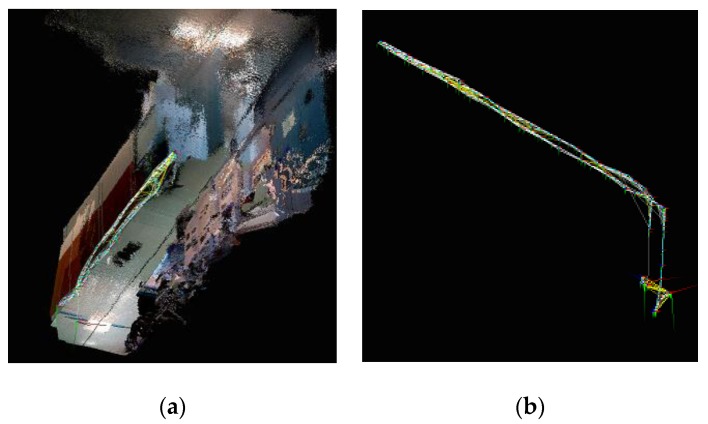
(**a**) 3D Map made by a RGB-D camera of the UAV; (**b**) navigation trajectory of the UAV.

Figure 6a,b show the mapping information and navigation trajectory generated using an RGB-D camera and aerial robot. Table 3 lists the location errors and the computing speed based on the robot’s navigation number within the experimental environment.

This study conducted location recognition using the aerial robot’s IMU sensor and visual odometry to generate a map using the aerial robot and RGB-D sensor. This study verified that aerial robot was not affected by geographic features, unlike the mobile robot, and thus reduced location recognition errors. However, owing to the large amount of computations, map generation took a long time, at a speed of about 3 fps. Even though the processing speed using the 2D LRF SLAM is relatively high, there were instances in which obstacle recognition issues occurred. Considerably large location recognition errors also occurred during map generation when using the mobile robot, owing to geographic features such as the slope. To overcome these shortcomings, this study used an aerial robot, which has a relatively free range of motion, and an RGB-D sensor that can obtain 3D depth information. However, real-time location recognition was difficult when using 3D map information to detect obstacles of various features because of the large amount of computation required. To resolve these issues, this study proposes a 2.5D mapping method, which incorporates a 2D map drawn through the 2D LRF with the height information incorporated in the 3D map information drawn by the RGB-D camera installed in the aerial robot.

## 3. 2.5D Elevation Mapping

An elevation map is a 2.5D map that incorporates height information into the 2D map. Recently, elevation maps have been used to reduce amount of information and improve the processing speeds of the algorithm by converting the height information of 3D maps to 2D maps for the robot’s SLAM. In [42], elevation mapping that uses imagery was proposed. However, as large amount of pixel data are used when using imagery, generating elevation map in real-time navigation becomes difficult. Further, when using a 3D laser scanner to obtain 3D information, in many instances, the sensor price is high and it is difficult to generate a 2D elevation map during real-time navigation [43,44]. Another method involves installing a sonar sensor and distance sensor in an aerial robot and generating the map by stacking 2D scan information based on height [14]. In such an instance, height measurement error of the sensor leads to errors in map information. Accordingly, this study used RGB-D sensor (Xtion from ASUS), which is a low-cost RGB-D sensor, to generate an elevation map. In addition, this study used 2D LRF to increase the accuracy of the robot’s location estimation and 2D matching of the elevation map.

**Figure 7:**
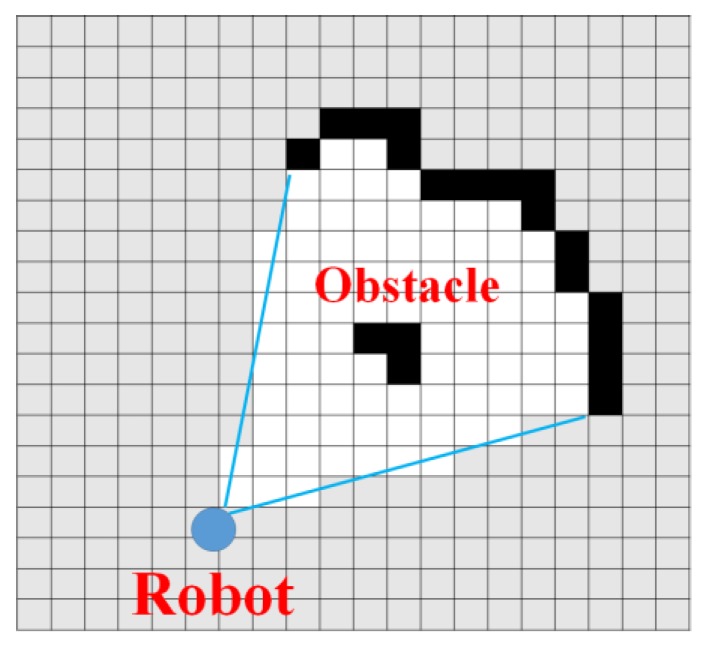
Occupancy grid map.

This study used an occupancy grid map to generate the 2D map [45]. An occupancy grid map is a grid block map, which is the most typical map concept used for generating 2D maps. Each block expresses geographic features and obstacles surrounding the robot detected by the robot’s sensor in white and black. Figure 7 is an image that simply expresses an occupancy grid map.

Figure 7 depicts laser-range sensor information as an occupancy grid. The black blocks, white blocks and grey blocks respectively indicate occupied area, unoccupied area where navigation is possible, and unexplored area.

Described below is the process that generates the 2D grid occupancy map, estimates the height of the obstacle from a sensor data, and incorporates the height information into the 2D grid map [46,47].

As shown in Equation (13), occupancy grid maps are determined by multiplying the probability value of each cell.

(13) $$P\left(M|X_{0:t},Z_{0:t}\right)=\underset{n}{\prod }p(m_{n}|X_{0:t},Z_{0:t})$$

Here, $X_{0:t}$ is the robot’s location based on time, and $Z_{0:t}$ is the sensor value at each location. The $m_{n}$ value of each block is calculated using Equations (14) and (15).

(14) $$p\left(m_{n}|X_{0:t},Z_{0:t}\right)=1-\frac{1}{1+e^{l_{t,n}}}$$

(15) $$l_{t,n}=l_{t-1}+\log\frac{p(m_{n}|x_{t},z_{t})}{1-p(m_{n}|x_{t},z_{t})}-\log\frac{1-p\left(m_{n}\right)}{p\left(m_{n}\right)}$$

Here, $p(m_{n})$ is the previous value of the block value $m_{n}$, and the value $p\left(m_{n}|X_{0:t},Z_{0:t}\right)$ is the probability value of the block value $m_{n}$ calculated using the sensor value $Z_{0:t}$ and the robot’s location value $X_{0:t}$ based on time. To incorporate height information into the generated 2D grid map, this study used a KF-type formula given in Equation (16) to estimate obstacle height.

(16) $$\mu_{0:t}=\frac{\sigma_{t}^{2}\mu_{o:t-1}+\sigma_{0:t-1}^{2}h_{t}}{\sigma_{0:t-1}^{2}+\sigma_{t}^{2}}$$

(17) $$\sigma_{0:t}^{2}=\frac{\sigma_{0:t-1}^{2}\sigma_{t}^{2}}{\sigma_{0:t-1}^{2}+\sigma_{t}^{2}}$$

Here, $h_{t}$ is the measured value of the sensor, and $\sigma_{t}^{2}$ is the variance of noise value included in the measured value of the sensor. The above formula was used to calculate the height value $\mu_{0:t}$ and the variance of the height error value $\sigma_{0:t}^{2}$.

Next, an elevation map is generated through the probability model described below to include the height values of Equation (16) in map M.

(18) $$P\left(M|X_{0:t},Z_{0:t},\mu_{0:t},\sigma_{0:t}^{2}\right)=\underset{n}{\prod }p(m_{n}|X_{0:t},Z_{0:t},\mu_{0:t},\sigma_{0:t}^{2})$$

(19) $$p\left(m_{n}|X_{0:t},Z_{0:t},\mu_{0:t},\sigma_{0:t}^{2}\right)=1-\frac{1}{1+e^{l_{t,n}}}$$

(20) $$l_{t,n}=l_{t-1}+\log\frac{p(m_{n}|x_{t},z_{t,}\mu_{0:t},\sigma_{0:t}^{2})}{1-p(m_{n}|x_{t},z_{t},\mu_{0:t},\sigma_{0:t}^{2})}-\log\frac{1-p\left(m_{n}\right)}{p\left(m_{n}\right)}$$

A 2D map (grid map) was generated as a map M. The time location $X_{0:t}$ of the robot and the 2D LRF sensor value $Z_{0:t}$ entered in the location were used. A KF-type formula was used to add height information along with the calculated height value and variance value, ${\;\mu }_{0:t}\;\mathrm{and}\;\sigma_{0:t}^{2}$. Moreover, when navigating with a mobile robot, the robot must be able to recognize slopes as navigable areas and stairs as obstacles by distinguishing between the two. The robot obtains height value of the obstacle by using the height value $\mu_{0:t}$ and the previous height value $\mu_{0:t-1}$, which are calculated to determine slopes and stairs. As shown in Equation (21), it recognizes obstacles when the threshold angle ϕ is exceeded, and it ignores the instances equal to or less than the threshold.

(21) $$f\left(x\right)=\{\begin{array}{cc} \mathrm{Recognition}, & \mu_{0:t}-\mu_{0:t-1}>\emptyset \\ \mathrm{Ignore}, & \mu_{0:t}-\mu_{0:t-1}\leq \emptyset \end{array}$$

An aerial robot was taken off to generate an elevation map. Data of the RGB-D sensor and the 2D LRF sensor are gathered to calculate the robot’s current location and correct location recognition errors. After that, scan matching with LRF scan information was used to correct errors in location estimation. With obstacle height calculated with RGB-D depth information, the slope of the obstacle was also calculated. When the slope of the obstacle exceeds the threshold of a critical angle, it is recognized as an obstacle, while ignored when it does not. In addition, another process was needed to project the height information obtained with an RGB-D sensor in the LRF generated grid map. It is difficult to generate 2D map information when using only a 3D RGB-D sensor, while the 3D RGB-D sensor used in this study was not suitable in scan matching because of its small angle of view. To overcome this shortcoming, a 2D grid map was generated through the LRF sensor, and the height information obtained by the RGB-D sensor was mapped onto the 2D map. 

Described below is the process using the height information measured from the RGB-D sensor to redefine probability value of the grid map, which was generated via the earlier 2D LRF sensor, and to map the 3D coordinates into 2D coordinates [48].

The *P_c_* of Equation (22) is the depth value entered through the RGB-D sensor.

(22) $$P_{c}={\left(x_{c},y_{c},z_{c}\right)}^{T}$$

The following is the estimated distance ($l_{p}$) of the depth pixels.

(23) $$l_{p}=\sqrt{x_{c}^{2}+y_{c}^{2}+z_{c}^{2}}$$

The following equations define the probability values s of each cell of the 2D grid map.

(24) $$P\left(s\left(l\right)\right)=P_{occ}\left(l\right)+\left(\frac{k}{\Delta l_{p}\sqrt{2\pi }}+0.5-P_{occ}\left(l\right)\right)e^{-\frac{1}{2}{\left(\frac{1-l_{p}}{\Delta l_{p}}\right)}^{2}}$$

(25) $$P_{occ}\left(l\right)=\{\begin{array}{cc} p_{min,}, & if\;0<l<l_{p}\\ 0.5, & if\;l>l_{p} \end{array}$$

Here, l and *k* are values of the distance and angle measured by the LRF sensor, respectively. The occupancy function $P_{occ}$ compares the distance measured through the RGB-D sensor and distance measured through the LRF sensor, and redefines the probability value *s* of each cell of the 2D grid map. If the distance measured through the RGB-D sensor is smaller than that through the existing LRF sensor, it is defined as a probability of 0.5. The reason for this is that a grid map is created as a navigable area if the probability value is higher or lower than 0, and as an unverified area if the probability value is 0. The function L(s(l)) updates the grid map through a log function and the probability value redefined above.

(26) $$L\left(s\left(l\right)\right)=\ln\left(\frac{P\left(s\left(l\right)\right)}{1-P\left(s\left(l\right)\right)}\right)$$

Finally, a 2.5D elevation map is generated by projecting object’s height information, which was calculated using the RGB-D sensor, onto the 2D grid map information obtained by the LRF sensor. 

**Figure 8:**
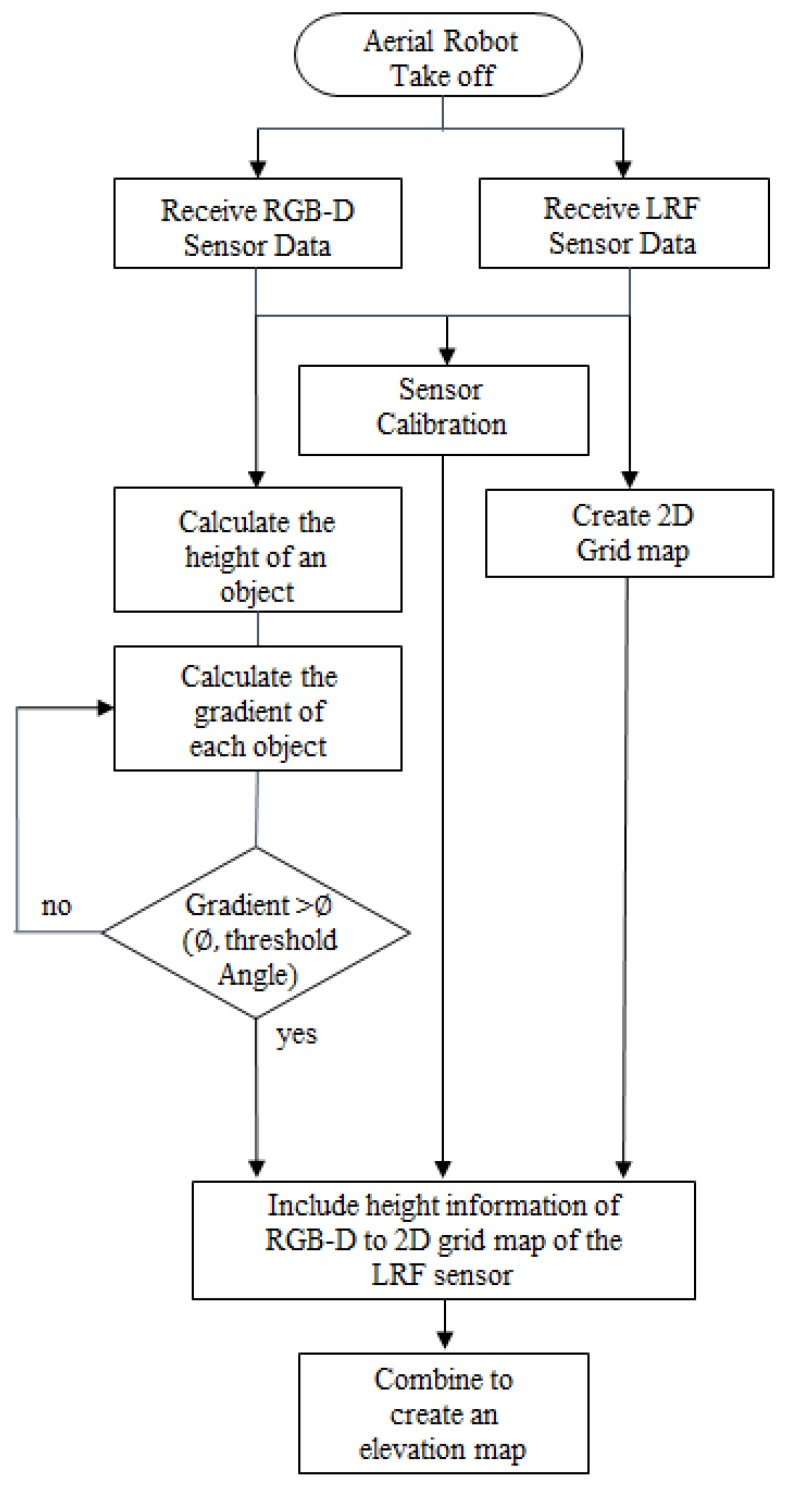
Flow chart for creating the 2.5D elevation map.

Figure 8 is a flow chart of the algorithm used for generating the 2.5D elevation map.

## 4. Experiments and Discussion

**Figure 9:**
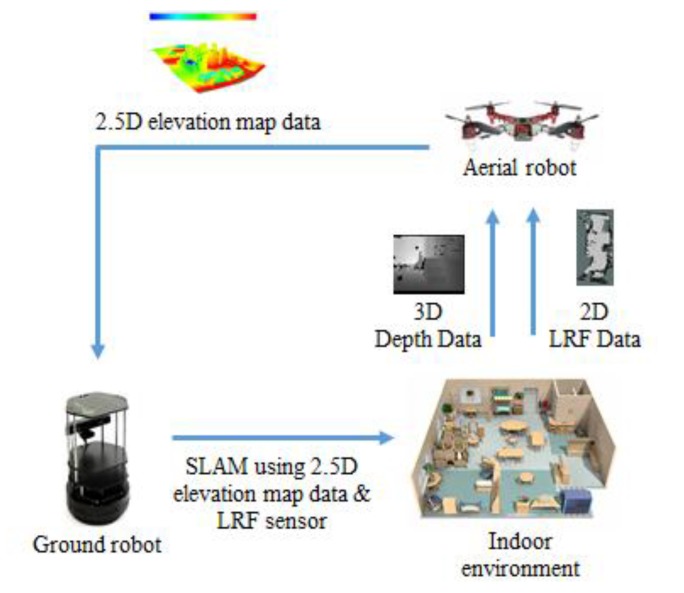
Relationship between the aerial robot and the ground robot in this study.

This study used the aerial robot of Figure 5 in an indoor environment that can explore vast area without being affected by geographic features for accurate and real-time location recognition, and built the 2.5D elevation map proposed in Section 3 as shown in Figure 9. Through a series of experiments, we checked the map building time and computing speed of the algorithm by comparing with the 3D map generated by the RGB-D sensor and proposed 2.5D elevation map. Furthermore, this study also intended to verify that the proposed 2.5D map could provide information on various obstacles through incorporating height information and comparing the result with that of the 2D map generated using an LRF sensor.

**Figure 10:**
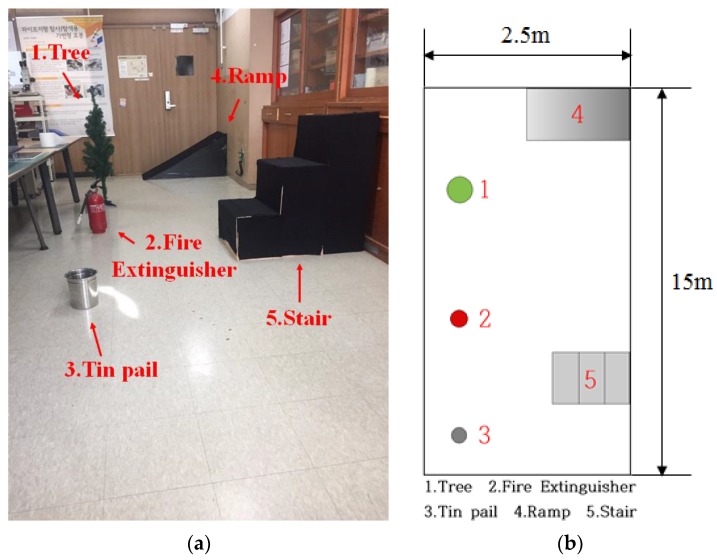
(**a**) Experiment environment; (**b**) layout of objects in the experimental environment.

To compare the accuracy of map generation and location recognition of the RGB-D SLAM, LRF SLAM, and the algorithm proposed in this paper, the robot navigated back and forth within a fixed distance (10 m) under the experimental environment shown in Figure 10a. After the navigation, error between the robot’s actual location and the estimated location was calculated. Moreover, as shown in Figure 10b, obstacles included a tree, fire extinguisher, tin pail, ramp, and stairs. 

**Figure 11:**
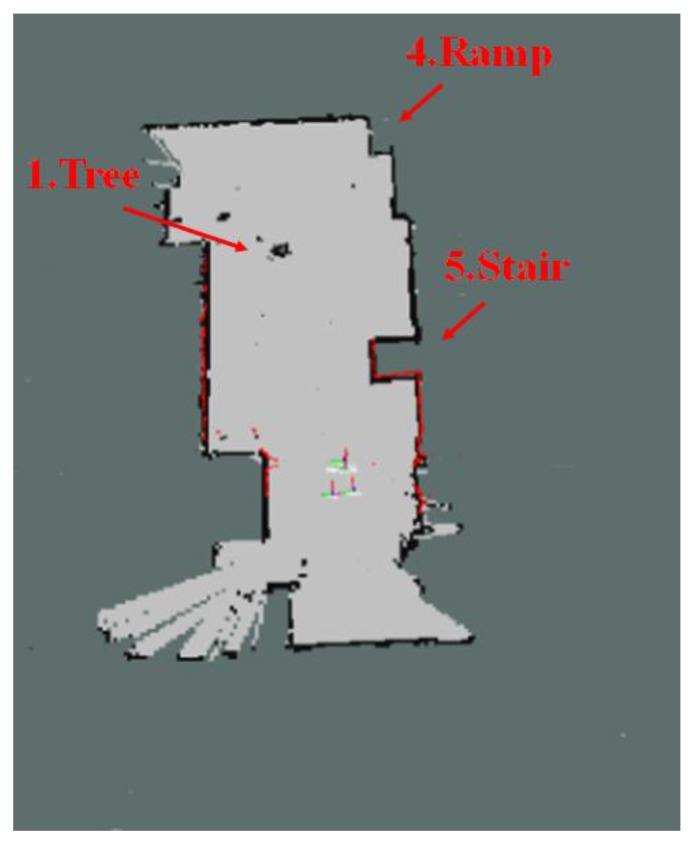
2D map built using the LRF sensor.

**Figure 12:**
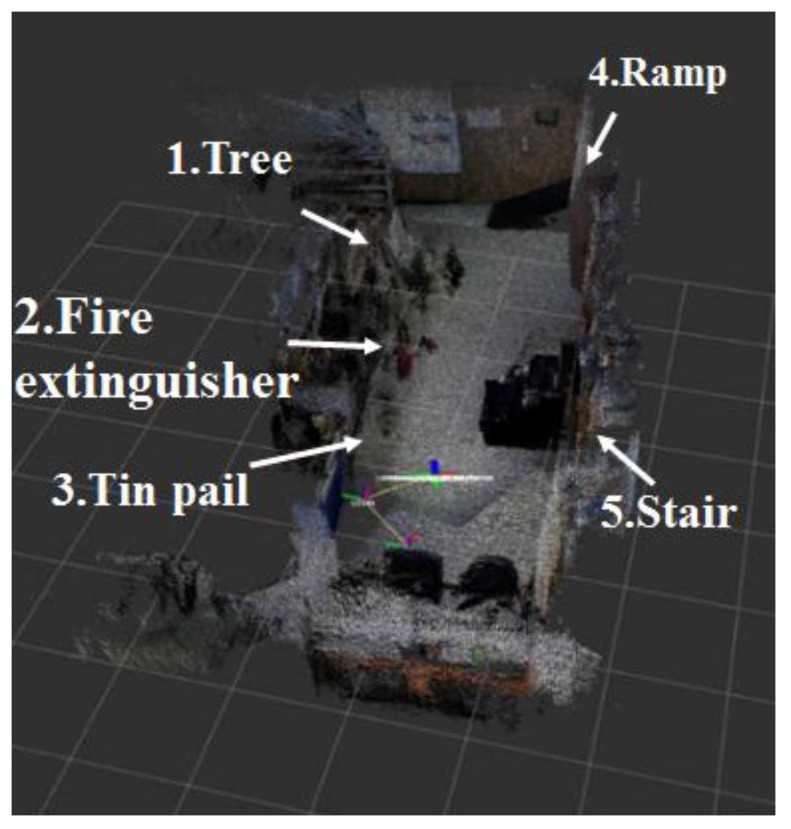
3D map created using RGB-D sensor of the aerial robot.

Figure 11 shows the map generated by using the 2D LRF sensors installed in the aerial robot, of which flying height was set as 1 m, and the results of the map generation show that the tree, stairs, and ramp that were located higher than the flying height of the aerial robot were recognized as obstacles, but the objects located lower than that were not recognized. Figure 12 represents the 3D map generated using the RGB-D sensors of the aerial robot, showing that it was possible to recognize even the ramp, stairs and objects of lower height, unlike the 2D LRF SLAM. Table 4 lists the location recognition errors and computing speeds of the 2D LRF SLAM and the RGB-D SLAM. It shows that the 2D LRF SLAM was faster in its average computing speed (26 fps), compared to the RGB-D SLAM (3–4 fps). The average location recognition errors of the 2D LRF SLAM and the RGB-D SLAM were 2.3 cm and 4.6 cm, respectively. 

**Figure 13:**
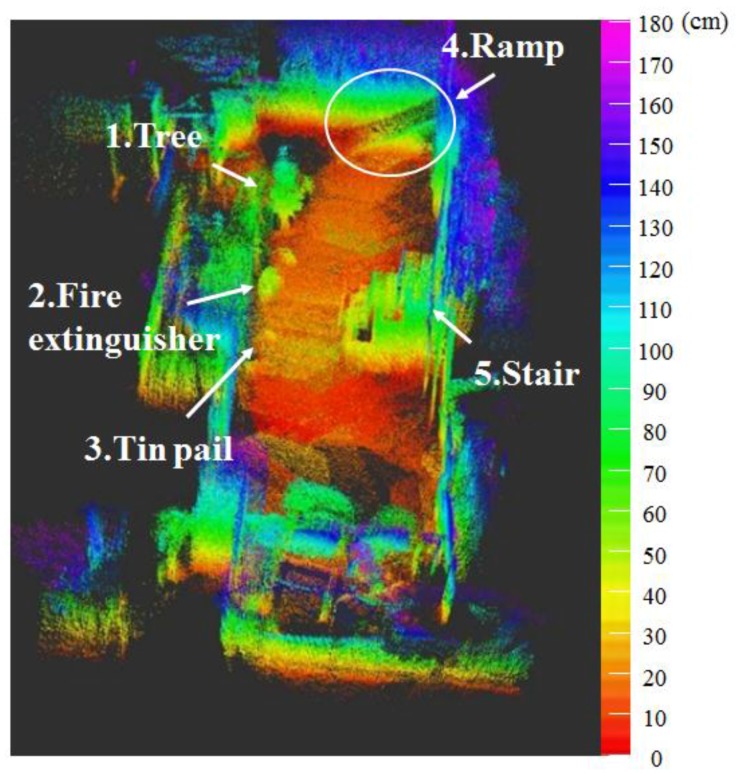
Height information of 3D map obtained using RGB-D sensors.

**Figure 14:**
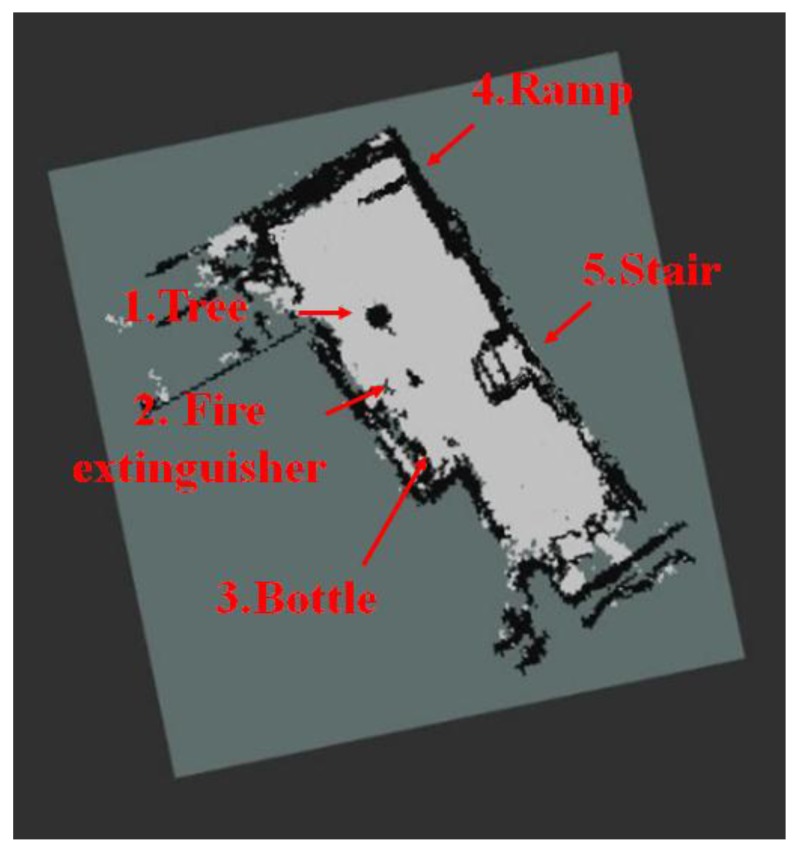
2D grid map information generated using 2D LRF.

Figure 13 and Figure 14 respectively show the height information of the 3D map built using the RGB-D sensors and the 2D grid map generated using the LRF sensor. 

**Figure 15:**
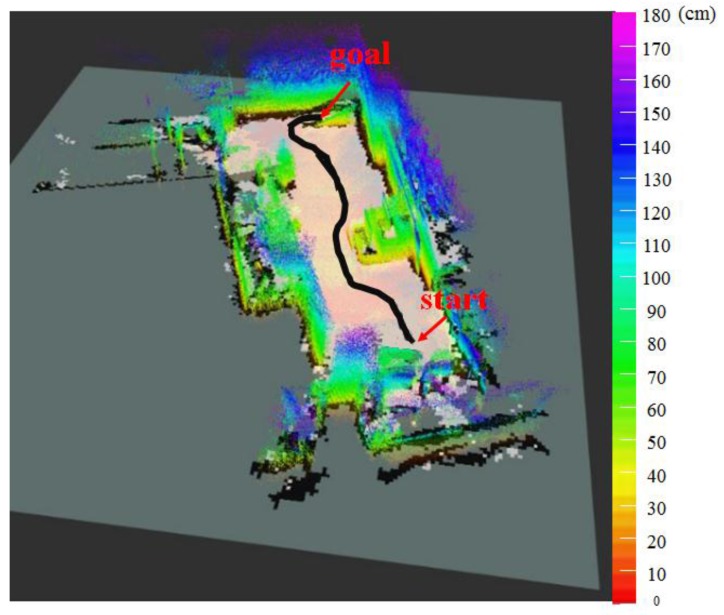
2.5D elevation map generated through mapping of height information of 3D map and 2D grid map.

Figure 15 shows the 2.5D elevation map generated with the proposed algorithm by combining the height information of Figure 13 and the 2D grid map of Figure 14. Table 5 represents the location recognition errors and computing speeds of the SLAM based on the proposed 2.5D elevation map. The SLAM-based 2.5D elevation map showed location recognition error of approximately 3 cm, similar to that of the 2D LRF-generated map, and a computing speed of approximately 19 fps, which is six times faster than that of the RGB-D SLAM-based map. 

**Figure 16:**
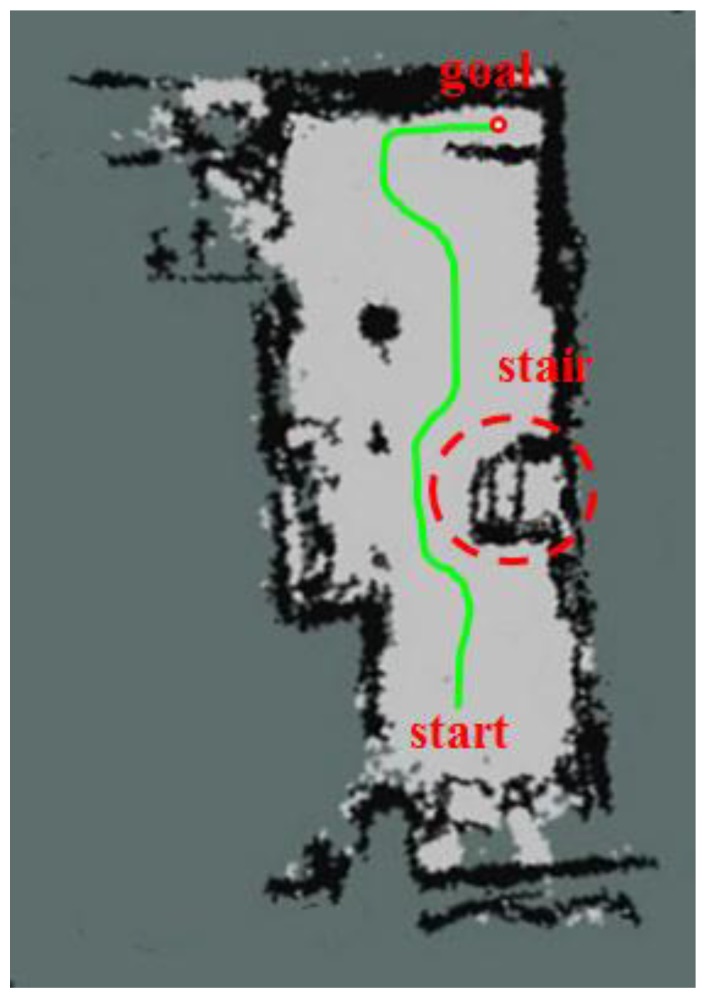
Mobile robot’s navigation results using the topographical information of elevation map on the 2D grid map.

Next, a navigation experiment was conducted to check whether tasks of avoiding the obstacles and travelling up the slope were possible when the mobile robot is using the proposed SLAM algorithm. The black line of Figure 15 shows the traveling trajectory of the mobile robot using the 2.5D elevation map. Figure 16 separately shows the grid map of the 2.5D elevation map so that the navigation results presented above can be more clearly. The red dot and green line respectively refer to the destination and actual navigation route traveled by the mobile robot.

The results of the navigation shows that the robot avoided and circumvented the lower parts of the stairs that were not recognized in the 2D map generated using the LRF sensor. The robot was also able to reach its destination by confirming navigable areas of the ramp and taking that route. 

If a moving obstacle enters the sensor’s working area when comparing the similarity between the current frame and the previous, presence of obstacle can be determined by using the 2D LRF sensor attached onto the mobile robot, but the obstacle cannot be recognized as the obstacle since the sensor is unable to recognize it as the most closest point during renewal of the map. However, even if the obstacle is moving but stays for at least 10 frames, it can be recognized as the obstacle and marked as impassable point on the renewed map.

We showed the results in the Table 4 and Table 5 as a single summary table, Table 6, to compare the performances of existing methodology and the one proposed in this paper. It did not show much difference regarding location recognition error compared to 2D LRF SLAM and RGB-D SLAM, but computing speed of the proposed method was about six times faster than that of the RGB-D SLAM. 

**Figure 17:**
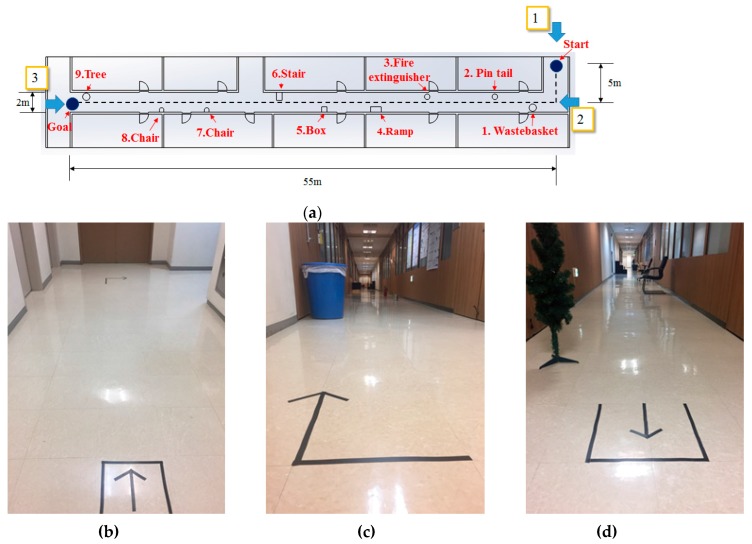
(**a**) Experimental environment layout with wide, complex indoor configuration; (**b**) view from 1; (**c**) view from 2; (**d**) view from 3.

In order to show expandability of the proposed localization method, we carried out the experiment in the indoors with more complicated shape, such as L-shape corridor. As shown in Figure 17, the experimental environment includes obstacles of various heights, including tree, chair, stairs, slope and fire extinguisher. The length of the moving path of the robot was 60 m and the path includes L-shape section, leading to the straight course. In order to measure the localization recognition error, we calculated the error as difference in the real location of the robot and the estimated value after running the robot from the starting point to the goal.

**Figure 18:**
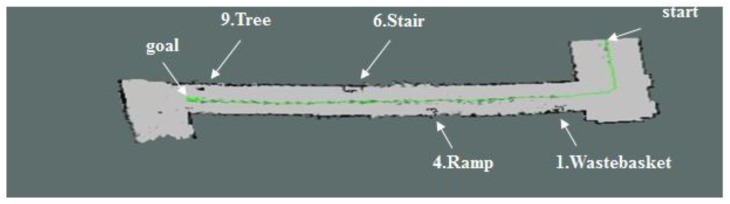
2D map built using the LRF sensor.

When the map was generating by using the 2D LRF as shown in Figure 18, there were obstacles that were detected and the ones that were not, depending on the flying altitude of the aerial robot. The aerial robot that worked at a height of 1 m detected tall obstacles (tree, upper stairs, upper part of the slope, garbage can), but not the lower ones. For the 2D LRF SLAM, location recognition error was about 2~3 cm and computing speed was about 25~30 FPS, which is quite fast. 

**Figure 19:**
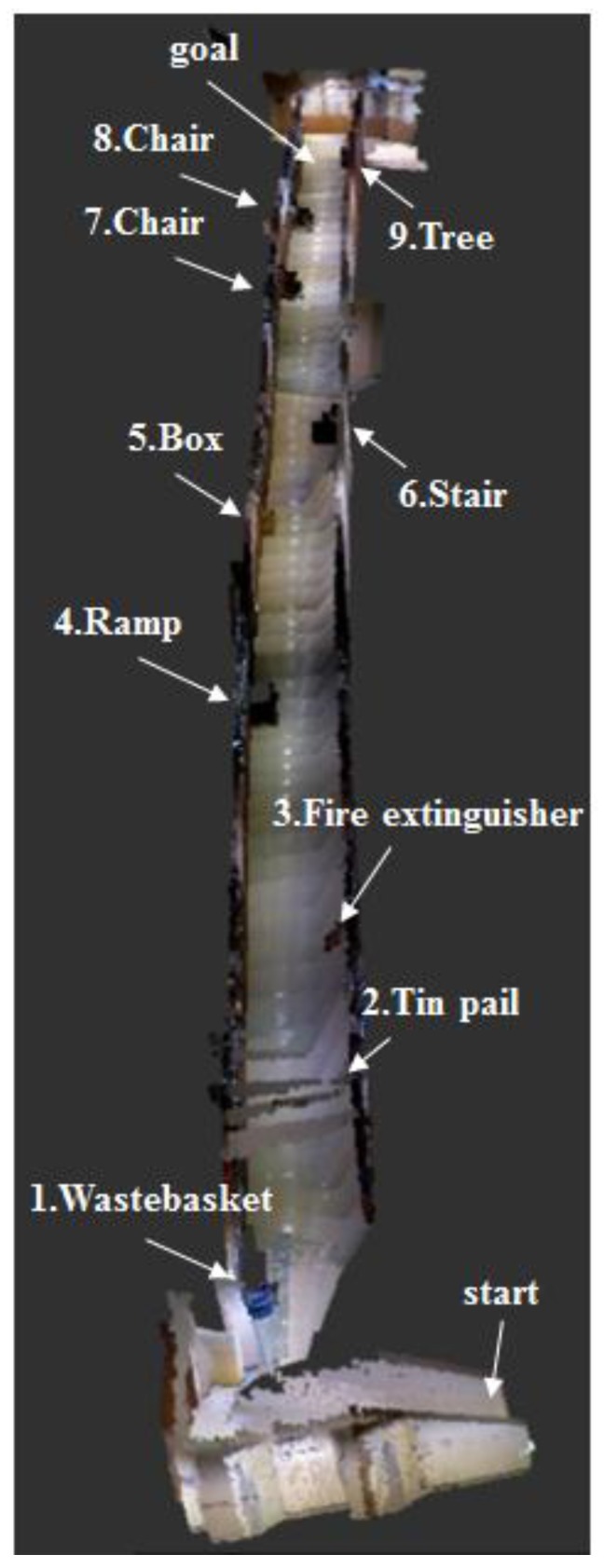
3D map created using the RGB-D sensor of the aerial robot.

When the 3D map was generated with the RGB-D sensor as shown in Figure 19, all obstacles were detected, regardless of the height. 3D RGB-D SLAM showed localization recognition error of about 3~4 cm, which was similar to that of the 2D LRF SLAM, but slow computing speed of about 2~3 FPS.

**Figure 20:**
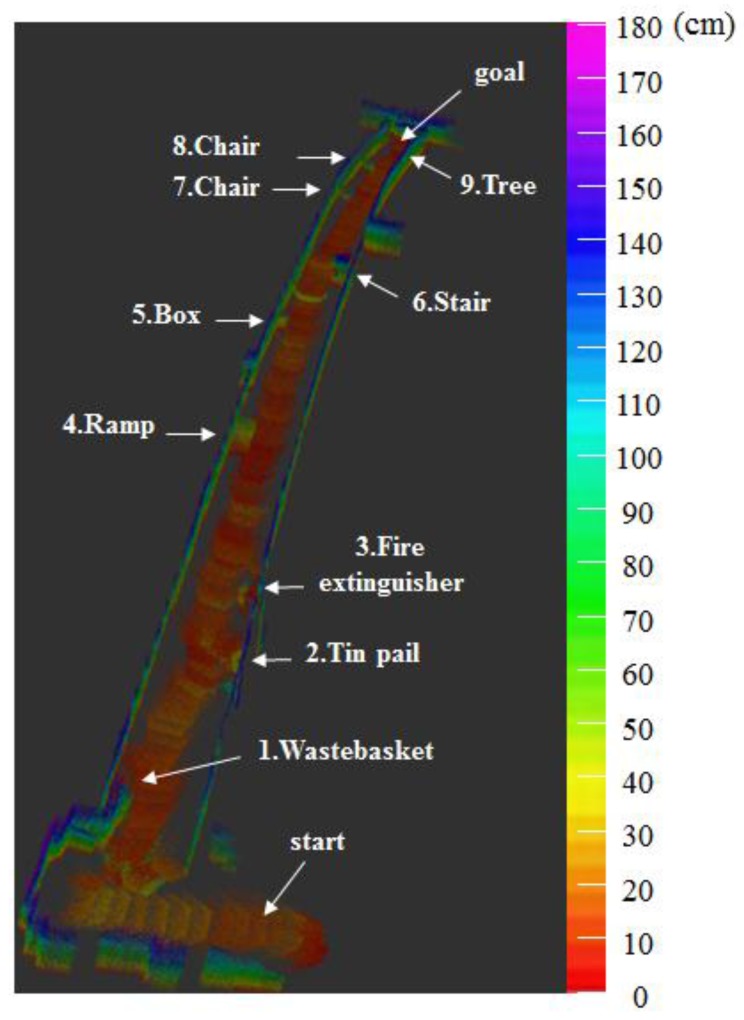
2.5D elevation map generated by the proposed method.

We generated the 2.5D elevation map as shown in Figure 20 on the experimental environment shown in Figure 17. Unlike the 2D map information shown in Figure 18, it could detect all obstacles, regardless of the height of the obstacle. Also, it could incorporate height information of the obstacle like the 3D map drawn by the RGB-D sensor as shown in Figure 19. As shown on Table 7, the 2.5D Elevation Map-based SLAM showed 2~3 cm location recognition error and the computing speed of approximately 19 fps, which is slower than that of 2D LRF SLAM (26 fps), but approximately seven times faster than that of the RGB-D SLAM. Therefore, according to Table 7, the methodology proposed by this paper showed that it maintains localization recognition errors and computing speed at a certain level, regardless of the size of the application area.

**Figure 21:**
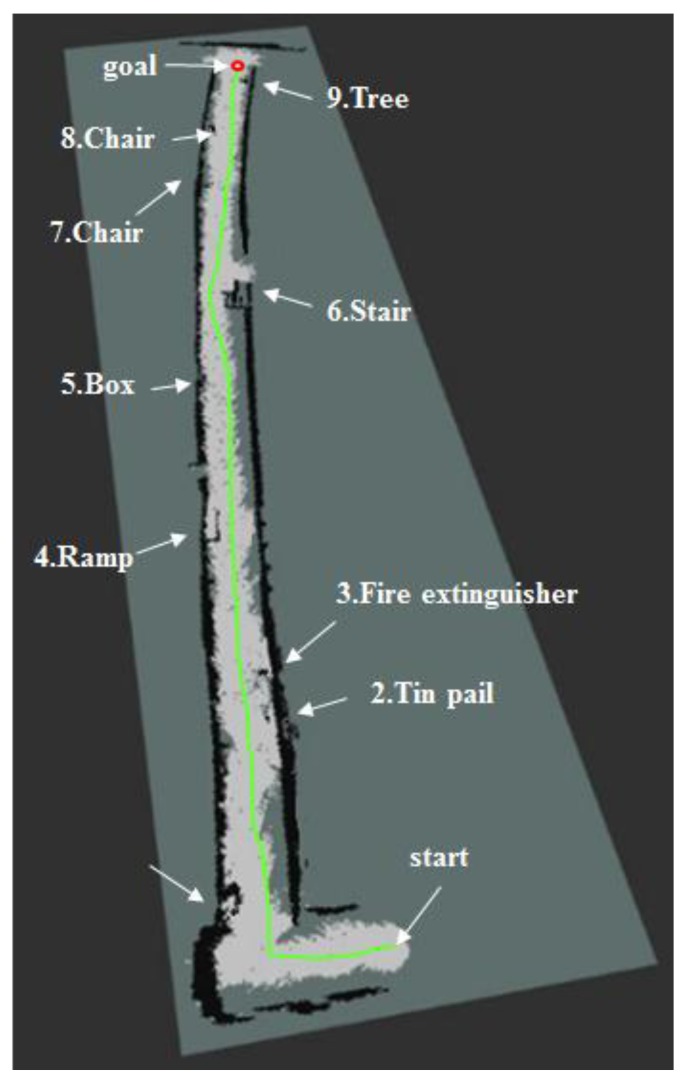
Mobile robot’s navigation results using the topographical information of elevation map on the 2D grid map.

Figure 21 shows the traveling trajectory of the mobile robot using the 2.5D elevation map. The red dot and green line respectively refer to the destination and the actual navigation route traveled by the mobile robot. The robot was able to reach its destination without colliding with obstacles. 

## 5. Conclusions

This paper proposes a mobile robot localization technique through the cooperation of UGV and UAV. A map was generated using an RGB-D sensor and a 2D LRF sensor installed in an aerial robot. The generated map was used for the mobile robot’s autonomous navigation and path generation. The existing 2D LRF SLAM is unable to detect certain obstacles owing to their height, while the RGB-D SLAM involves large amounts of data to be computed for 3D mapping, making the use of real-time algorithms difficult. To resolve these issues, this study simultaneously used both methods and proposed a new 2.5D elevation-mapping method that incorporates height information from 3D maps into a 2D map. 

The effectiveness of the proposed method was validated through a series of experiments. The proposed 2.5D map-based SLAM demonstrated similar location recognition accuracy to that of the 2D LRF SLAM. It was also shown to be able to recognize obstacles lower in height, such as the stairs and ramp that were not recognized by the 2D LRF SLAM. With its computing speed that is six times faster than that of the RGB-D SLAM, it sheds light on the increased feasibility of the real-time localization.

The results presented are encouraging and demonstrate the potential of collaborative robotic systems cooperating to provide accurate, real-time localization of mobile robots in SLAM generated maps. 

Future research is needed to generate a single, consolidated 2.5D map for larger indoor environments, such as multi-level buildings. By utilizing a number of aerial robots that are designed to discover various areas of larger indoor environments, each aerial robot may generate 2.5D maps through the proposed localization methodology. The generated maps could be combined to form a consolidated map covering the whole area of large indoor environment.

## Figures and Tables

**Table 1:** Location recognition errors according to the number of the mobile robot’s navigation in case of 2D LRF SLAM.

Navigation Trial	Location Recognition Error
1	32 cm
2	32 cm
3	31 cm
4	31 cm
5	27 cm
6	32 cm
7	28 cm
8	30 cm
9	33 cm
10	32 cm
Average	30.8 cm

**Table 2:** Location recognition errors according to the number of the mobile robot’s navigation in case of RGB-D SLAM.

Navigation Trial	Location Recognition Errors	Computing Speed (FPS)
1	5 cm	3
2	6 cm	3
3	8 cm	3
4	5 cm	4
5	7 cm	3
6	5 cm	3
7	9 cm	3
8	8 cm	4
9	6 cm	3
10	7 cm	4
Average	6.6 cm	3.3

**Table 3:** Location recognition errors and computing speed according to the number of the UAV’s navigation with RGB-D SLAM.

Navigation Trial	Location Recognition Error	Computing Speed (FPS)
1	2 cm	3
2	2 cm	2
3	4 cm	3
4	1 cm	4
5	3 cm	3
6	2 cm	3
7	1.6 cm	3
8	2 cm	3
9	1 cm	3
10	2 cm	3
Average	2.1 cm	3

**Table 4:** Comparison of 2D LRF SLAM and RGB-D SLAM.

Navigation Trial	Location Recognition Error		Computing Speed (FPS)	
	2D LRF	RGB-D	2D LRF	RGB-D
1	3 cm	4 cm	25	3
2	2 cm	5 cm	27	4
3	2 cm	5 cm	25	3
4	2 cm	5 cm	25	3
5	3 cm	5 cm	25	3
6	2 cm	4 cm	24	3
7	3 cm	4 cm	26	3
8	2 cm	5 cm	25	3
9	2 cm	5 cm	24	3
10	2 cm	4 cm	25	3
Average	2.3 cm	4.6 cm	25.1	3.1

**Table 5:** Location recognition errors and computing speeds of the proposed 2.5D elevation map-based SLAM.

Navigation Trial	Location Recognition Error	Computing Speed (FPS)
1	3 cm	19
2	2 cm	20
3	3 cm	19
4	3 cm	18
5	3 cm	19
6	3 cm	19
7	2 cm	20
8	3 cm	19
9	3 cm	19
10	3 cm	19
Average	2.8 cm	19.1

**Table 6:** Comparison of 2D LRF SLAM, RGB-D SLAM and the proposed method.

Experimental Results	Location Recognition Error (cm)			Computing Speed (FPS)		
	2D LRF SLAM	RGB-D SLAM	The Proposed Method	2D LRF SLAM	RGB-D SLAM	The Proposed Method
Average	2.3	4.6	2.8	25.1	3.1	19.1
Ratio	0.5	1	0.61	8.1	1	6.2

**Table 7:** Comparison of 2D LRF SLAM, RGB-D SLAM and the proposed method in experimental environment of Figure 16.

Navigation Trial	Location Recognition Error (cm)			Computing Speed (FPS)		
	2D LRF SLAM	RGB-D SLAM	2.5D Elevation SLAM	2D LRF SLAM	RGB-D SLAM	2.5D Elevation SLAM
1	3	4	3	25	2	19
2	2	3	2	27	3	18
3	3	4	3	27	2	19
4	2	4	3	25	2	18
5	3	3	3	30	4	18
6	2	4	3	25	3	19
7	3	4	2	24	2	22
8	2	3	3	28	2	19
9	3	5	3	24	3	18
10	3	4	3	25	2	19
Average	2.8	3.8	2.8	26	2.5	18.9
